# The role of microvessel density on the survival of patients with lung cancer: a systematic review of the literature with meta-analysis

**DOI:** 10.1038/sj.bjc.6600551

**Published:** 2002-09-23

**Authors:** A-P Meert, M Paesmans, B Martin, P Delmotte, T Berghmans, J-M Verdebout, J-J Lafitte, C Mascaux, J-P Sculier

**Affiliations:** Département de Médecine Interne et Laboratoire d'Investigation Clinique et d'Oncologie Expérimentale HJ Tagnon, Institut Jules Bordet, Bruxelles, Belgium; Data Centre, Institut Jules Bordet, Bruxelles, Belgium; Service d'Anatomo-Pathologie, Institut Jules Bordet, Bruxelles, Belgique; Service de Pneumologie et d'Oncologie Thoracique, CHU Calmette, Lille, France

**Keywords:** lung cancer, microvessel count, microvessel density, meta-analysis

## Abstract

In order to determine whether angiogenesis is a prognostic marker in lung cancer, we performed a systematic review of the literature to assess the prognostic value on survival of microvessel count in patients with lung cancer. Published studies were identified by an electronic search in order to aggregate survival results, after a methodological assessment using a quality scale designed by the European Lung Cancer Working Party. To be eligible, a study had to deal with microvessel count assessment in lung cancer patients on the primary site and to provide survival analysis according to microvessel count expression. Microvessel count has been assessed on surgical samples by immunohistochemistry using factor VIII in 14 studies, CD34 in 10 and CD31 in eight. Respectively 1866, 1440 and 1093 non-small cell lung cancer patients were considered. The overall median quality scores were respectively 52, 59 and 59% for studies assessing microvessel count via factor VIII, CD34 and CD31, without significant difference between studies evaluable or not for meta-analysis nor between studies with significant or non significant results. Seven ‘factor VIII’ studies, nine ‘CD34’ and seven ‘CD31’ provided sufficient data allowing a meta-analysis on survival and were evaluable for results aggregation. This showed that a high microvessel count in the primitive lung tumour was a statistically significant poor prognostic factor for survival in non small cell lung cancer whatever it was assessed by factor VIII (HR: 1.81; 95% CI: 1.16–2.84), CD34 (HR: 1.99; 95% CI: 1.53–2.58) or CD31 (HR: 1.80; 95% CI: 1.10–2.96). Variations in survival among the individual studies can be explained in addition to patients selection criteria by the heterogeneous methodologies used to stain and count microvessels: different antibody clones, identification of ‘hotspots’, Weidner or Chalkey counting method, cut-off selection. Microvessel count, reflecting the angiogenesis, appears to be a poor prognostic factor for survival in surgically treated non small cell lung cancer but standardisation of angiogenesis assessment by the microvessel count is necessary.

*British Journal of Cancer* (2002) **87**, 694–701. doi:10.1038/sj.bjc.6600551 www.bjcancer.com

© 2002 Cancer Research UK

## 

Lung cancer is the most common cause of death by malignancy in industrialised countries. Less than 15% of the patients will be cured and enjoy long-term survival. This poor prognosis can be modulated by characteristics related to the patient or the tumour. These prognostic factors can be used for different purposes such as a better understanding of the natural history of the disease or the identification of homogeneous patient's populations with a similar outcome profile. Some independent clinical and biological predictors have been identified for predicting survival (Paesmans and Sculier, 1998): for resectable non small cell lung cancer (NSCLC) age, performance status and TNM stage (Strauss, 1997). Among routine biological factors, serum lactate dehydrogenase, white blood cell and neutrophil count have been shown to significantly predict survival in NSCLC (Kanters *et al*, 1995). Recent developments in cytogenetic and molecular biology have provided new ways to analyse prognosis. Biological substaging using molecular markers in a risk stratification strategy has been proposed. Tumour suppressor genes, proto-oncogenes, markers of proliferation and angiogenesis are some of the different research tools.

Angiogenesis is the formation of new blood vessels from the endothelium of the existing vasculature. These new capillaries arise from pre-existing capillaries or venules and represent the consequence of the growth of columns of aligned endothelial cells. Adjacent columns contact to form loops, which then develop a lumen. Neo-angiogenesis is fundamental in tumour growth, progression and metastases and there is now experimental evidence to indicate that tumour growth is dependent on angiogenesis (Folkman, 1990). After a new tumour has attained a small size of 1–2 mm, further growth and expansion of the tumour require the induction of new blood vessels. Although this angiogenesis alone is not sufficient for developing metastases, new blood vessel formation increases the opportunity for malignant cells to enter the blood stream and thus the development of metastases (Weidner *et al*, 1991). Newly formed capillaries are permeable because of fragmented basement membranes, making them more accessible to errant tumour cells (Weidner *et al*, 1992).

Tumour angiogenesis is a complex multifactor process involving growth factor and extracellular matrix enzymes. A variety of proteins such as the vascular endothelial growth factor, the platelet-derived endothelial cell growth factors and the basic fibroblast growth factor released by tumour and stroma cells have been recognised to be potent inducers of angiogenesis (Bikfalvi *et al*, 1997; Ishikawa *et al*, 1989; Ferrara, 2000).

Recent evidence suggests that tumour angiogenesis is associated with patient outcome in a number of malignancies. Microvessel density seems to be an important prognostic indicator in lung cancer (Fontanini *et al*, 1995; Giatromanolaki *et al*, 1997) although some studies have not found microvessel count to be predictive for survival (Chandrachud *et al*, 1997; Pastorino *et al*, 1997). Currently, different antibodies to three endothelial cell antigens can be used to visualise the tumour blood vessels by immunohistochemistry: factor VIII antigen or von Willebrand's factor is involved in platelet adhesion and aggregation; CD31 or PECAM 1 (platelet/endothelial cell adhesion molecule) is associated with platelet adhesion in inflammation, wound healing, trans-endothelial cell migration and cell migration; CD34 is involved in leukocyte adhesion and endothelial cell migration during angiogenesis.

In order to determine whether microvessel count is a prognostic factor for survival in lung cancer patients, we performed a systematic review of the literature with methodological assessment.

## MATERIALS AND METHODS

### Publication selection

To be eligible for this review, trials had to deal with lung cancer only, to evaluate the correlation between microvessel count and survival, to measure the microvessel count in the primary tumour (not in metastatic tissue or in tissue adjacent to the tumour) and to be published as a full paper in the English or French language literature. Abstracts were excluded from this analysis because of insufficient data to apply the scoring system and to evaluate the methodological quality of the trial.

Articles were identified by an electronic search on Medline using the keywords lung neoplasms, CD31, PECAM-1, CD34, factor VIII, angiogenesis, neoangiogenesis, angiogenic factor, neovascularisation, microvessel, vessel density, vascular density or microvascular density. The bibliographies reported in all the identified studies were used for completion of the trials search. When authors reported, in several publications, on the same patients populations, only the most recent or complete study was included into the analysis, in order to avoid overlapping between cohorts. The search ended on September 2001.

### Methodological assessment

In order to assess the methodology, each trial was read and scored according to the ELCWP (European Lung Cancer Working Party) scale by nine investigators (including six physicians, one pathologist, one biologist and one biostatistician). Consensual agreement on the scores attributed to each item for each trial was obtained during meetings where the participation of many readers was a guarantee for the correct interpretation of the articles. The scoring system used in this literature review has already been described in one of our prior systematic reviews (Steels *et al*, 2001). The overall score assessed many dimensions of methodology, grouped in four main categories: the scientific design, the description of the laboratory methods used to quantify MVC, the generalisability of the results and the analysis of the study data. Each category had a maximal score of 10 points with an overall maximal theoretical score of 40 points. The final scores were expressed as percentages, higher values reflecting a better methodological quality. Studies included in the systematic review were called ‘eligible’ and those providing sufficient data for the meta-analysis ‘evaluable’.

### Statistical methods

A study was considered as significant if the *P* value for the statistical test comparing survival distributions between the groups with and without high microvessel count was <0.05 in univariate analysis. The study was called ‘positive’ when a high microvessel count was identified as a significant favourable prognostic factor for survival. The study was called ‘negative’ if the same characteristic was associated with a significant detrimental effect on survival. Finally, a study was called ‘not significant’ if no statistical difference between the two groups was detected.

The association between two continuous variables was measured by the Spearman ranks correlation coefficient. Mann–Whitney test was used to compare the distribution of the quality scores according to the value of a binary variable.

If it was possible, we dichotomised the variable MVC by using the observed median.

For the quantitative aggregation of survival results, we measured the impact of microvessel count on survival by the hazard ratio (HR) between the two survival distributions. For each trial, this HR was estimated by a method depending on the data provided in the publications. The most accurate method consisted of calculating the estimated HR and its standard error using two of the following parameters: the HR point estimate, the logrank statistic or its *P* value, the O-E statistic (difference between numbers of observed and expected events) or its variance. If those data were not available, we looked for the total number of events, the number of patients at risk in each group and the logrank statistic or its *P* value allowing calculation of an approximation of the HR estimate. Finally, if the only available data were in the form of graphical representations of the survival distributions, we extracted from them survival rates at some specified times in order to reconstruct the HR estimate and its variance, with the assumption that the rate of patients censored was constant during the study follow-up (Parmar *et al*, 1998). If this last method was used, three independent persons read the curves to reduce the imprecision in the reading variations. The individual HR estimates were combined into an overall HR using Peto's method (Yusuf *et al*, 1985), which consisted of using a fixed effect model assuming homogeneity of the individual true HRs. This assumption was tested by performing χ^2^ tests for heterogeneity. If the assumption of homogeneity had to be rejected, we used a random-effects model as a second step. By convention, an observed HR >1 implied a worse survival for the group with a high microvessel count. This pejorative impact of angiogenesis on survival was considered as statistically significant if the 95% confidence interval for the overall HR did not overlap 1.

## RESULTS

### Studies selection and characteristics

Twenty-one studies detecting MVC by factor VIII were selected. Seven of the articles (Angeletti *et al*, 1996; Fontanini *et al*, 1996, 1997b, 1998a; Harpole *et al*, 1996; Takanami *et al*, 1999; D'Amico *et al*, 2000) were excluded because identical cohorts of patients were included in other selected publications. In the 14 remaining eligible studies (Macchiarini *et al*, 1994; Yamazaki *et al*, 1994; Fontanini *et al*, 1995; Mattern *et al*, 1995; Chandrachud *et al*, 1997; Giatromanolaki *et al*, 1997; Takanami *et al*, 1997; Duarte *et al*, 1998; Imoto *et al*, 1998; Aikawa *et al*, 1999; D'Amico *et al*, 1999; Ohta *et al*, 1999; Sheng *et al*, 2000; Yano *et al*, 2000), published between 1994 and 2000, the total number of patients was 1866 ranging from 28 to 408. The main characteristics of these 14 studies are shown in Table 1

**Table 1:**
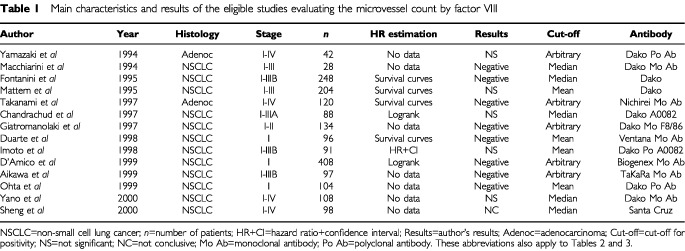
Main characteristics and results of the eligible studies evaluating the microvessel count by factor VIII

. Twelve of them dealt with NSCLC whatever the histologic subtype considered and two with adenocarcinoma only. Ten studies concerned only limited disease and four all stages (I to IV). Different antibodies were used to assess factor VIII positivity.

CD34 was used in 13 studies. Three of the articles (Lucchi *et al*, 1997; Fontanini *et al*, 1998b; Cox *et al*, 2000) were excluded because identical cohorts of patients were included in other publications. In the 10 remaining eligible studies (Fontanini *et al*, 1997a; Matsuyama *et al*, 1998; Shibusa *et al*, 1998; Dazzi *et al*, 1999; Cagini *et al*, 2000; Takanami *et al*, 2000; Yano *et al*, 2000; Cox *et al*, 2001; Liao *et al*, 2001; Offersen *et al*, 2001), published between 1997 and 2001, the total number of included patients was 1440 ranging from 44 to 407 patients by trial. The main characteristics of these 10 eligible studies are shown in Table 2

**Table 2:**
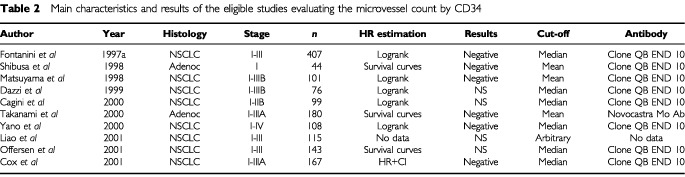
Main characteristics and results of the eligible studies evaluating the microvessel count by CD34

. Eight of them dealt with NSCLC whatever the histologic subtype considered and two with adenocarcinoma only. Nine studies concerned only limited stage disease and one, all stages. Most of the time, clone QB-END 10 monoclonal antibody was used to assess CD 34 immunoreactivity.

In terms of CD31 detection, 18 studies were selected. Ten were excluded (Giatromanolaki *et al*, 1996a,b, 1997, 2000a,b; Koukourakis *et al*, 1997, 1999, 2000a,b; Kakolyris *et al*, 1999) because identical cohorts of patients were used in other selected publications. In the eight remaining eligible studies (Apolinario *et al*, 1997; Kawaguchi *et al*, 1997; Pastorino *et al*, 1997; Duarte *et al*, 1998; Ohta *et al*, 1999; O'Byrne *et al*, 2000; Han *et al*, 2001; Hasegawa *et al*, 2001), published between 1996 and 2001, the total number of included patients was 1093 ranging from 15 to 515 patients by trial. The main characteristics of the eight studies eligible for the systematic review are shown in Table 3

**Table 3:**
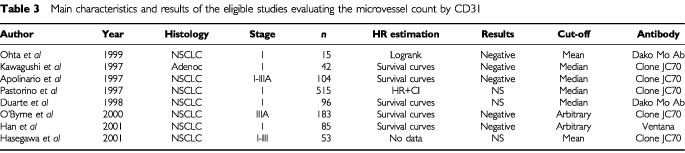
Main characteristics and results of the eligible studies evaluating the microvessel count by CD31

. Seven of them dealt with NSCLC whatever the histologic subtype and one with adenocarcinoma only. Seven studies concerned only limited stage disease and one all stages diseases. In five studies, JC70 monoclonal antibody was used to detect CD31.

For the three antibodies, immunoreactivity was always assessed on surgical samples.

### Studies results reports

When factor VIII was used to assess MVC, eight studies reported ‘negative’ results, five were not significant and one was not conclusive. Seven studies were evaluable for meta-analysis.

Looking at the survival results in the studies dealing with CD34, six studies were ‘negative’ and four were not significant. Nine studies were evaluable for meta-analysis.

When CD31 was detected, five studies were ‘negative’ and three not significant. Seven were evaluable for meta-analysis.

### Quality assessment

Concerning ‘factor VIII’ studies, the overall quality score ranged from 36.2% to 72.9% with a median of 52.4%. The ‘design’ subscore had the lowest value (median: 30%). There was no statistically significant quality difference between evaluable and non-evaluable studies for meta-analysis (median overall scores: 68.1% *vs* 49.6%, *P*=0.07). No statistically significant quality difference was shown between significant and non-significant trials (median overall scores 49.7% *vs* 56.9%, *P*=0.73).

For ‘CD34’ studies, the overall quality score ranged from 43.3% to 76.3% with a median of 59.3%. The ‘design’ subscore had the lowest median value (40%). No statistically significant quality difference was shown between the significant and the non-significant trials (median overall scores 63.6% *vs* 59.2%, *P*=0.67).

For ‘CD31’ studies, the overall quality score ranged from 38.9% to 72.9% with a median of 59.5%. The ‘design’ subscore had the lowest median value (45%). No statistically significant quality difference was shown between the significant and the non-significant trials (median overall scores 53.2% *vs* 69.9%, *P*=0.17).

There was no significant correlation between quality scores and the number of patients included in the studies or with the date of publication of the studies.

### Meta-analysis

The absence of significant quality difference between significant and non-significant studies allowed us to perform a quantitative aggregation of the survival results.

**Figure 1:**
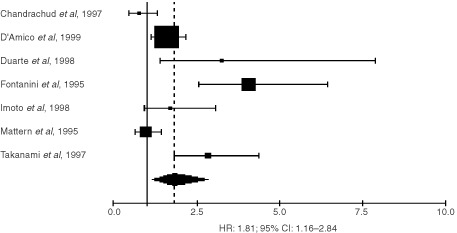
Results of the meta-analysis of the studies using factor VIII. HR>1 implies a survival disadvantage for the group with a high microvessel count. The square size is proportional to the number of patients included in the study. The centre of the lozenge gives the combined HR for the meta-analysis and its extremities the 95% confidence interval.

**Figure 2:**
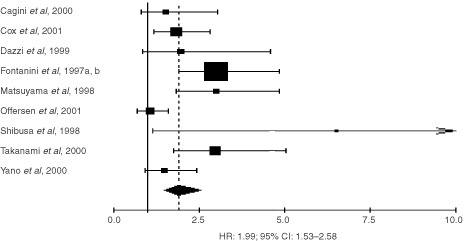
Results of the meta-analysis of the studies using CD34.

**Figure 3:**
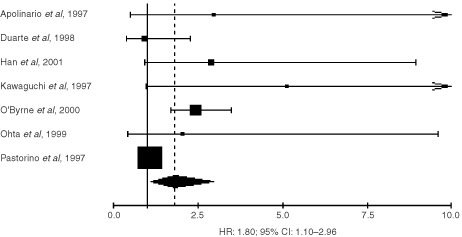
Results of the meta-analysis of the studies using CD31.

Among the 32 trials eligible for the systematic review evaluating MVC with factor VIII, nine could not be included in the meta-analysis due to insufficient data to estimate the HR or because data concerned only some subgroups of patients. The hazard ratios of the 23 evaluable studies were calculated by one of the three methods reported in the Materials and methods section. Hazard ratio and 95% confidence intervals were published in three trials. They were approximated from the logrank statistic and the number of events in eight studies. Finally, the HR and its variability had to be extrapolated from the graphical representations of the survival distributions in the 12 others. With a fixed-effect model, the HR was 1.71 (95% CI: 1.44–2.04) for factor VIII studies, 1.95 (95% CI: 1.65–2.30) for CD34 studies and 1.40 (95% CI: 1.17–1.63) for CD31 studies. However, the test of heterogeneity was significant for factor VIII (*P*<0.001), CD34 (*P*=0.02) and CD31 studies (*P*=0.004). Thus, we calculated the HR using a random-effects model and obtained a value which was statistically significant for factor VIII HR: 1.81 (95% CI: 1.16–2.84) (Figure 1

), for CD34 1.99 (95% CI: 1.53–2.58) (Figure 2

) and for CD31 HR: 1.80 (95% CI: 1.10–2.96) (Figure 3

).

## DISCUSSION

In this systematic review, by pooling all the studies comparing survival of lung cancer patients according to the angiogenic activity of the tumour, as expressed by the MVC, we show that a high MVC is a poor prognosis factor for survival in surgical NSCLC whatever the antibody used for assessment of the vessel count. This observation is potentially important for prognostic reasons and treatment purposes. Angiogenesis assessment might be useful not only in stratifying patients for different (adjuvant) treatment regimens but also in predicting their response to chemotherapy (Koukourakis, 2001; Mattern, 2001), to anti-angiogenic therapies and identifying the precancerous lesions (Pazouki *et al*, 1997).

It is also to be noted that the trials published on the topic concerned NSCLC treated, at least, by surgery; we could thus not extrapolate our results to metastatic NSCLC or small cell lung cancer.

To perform the meta-analysis, we have used a methodology similar to previous systematic reviews of our group on the treatment of lung cancer (Meert *et al*, 1999) but adapted to the field of biological prognostic factors (Steels *et al*, 2001). The absence of statistically significant difference in quality score between significant and non-significant publications allowed us to perform a quantitative aggregation of the individual trials results.

Our approach does not however prevent all potential biases. We restricted our review to articles published in French or English language because other languages such as Japanese were not accessible to the readers. This selection could favour the positive studies that are more often published in English while the negative ones tend to be more often reported in native languages (Egger *et al*, 1997). Another possible source of confusion is the use of a same cohort of patients in different publications. It might be difficult to avoid the same patients being included more than once in the meta-analysis. We have excluded publications where it seemed to be the case after writing to some of the authors in order to have more information on patients' cohort, a procedure that was unsuccessful: we had only one partial response. Harris confirmed that there were an overlapping of the same patients in the series of Giatromanolaki, Koukourakis *et al* The method of extrapolation of HRs also needs to be discussed. When HRs were not reported by the authors, they were calculated from the data reported in the article and, if not available, extrapolated from the survival curves, implying assumptions on the censoring process. This approach might also have been associated with errors due to imprecision in the reading, although three independent persons read the curves to reduce the reading variation.

Our review took into account only fully published studies. We did not look for unpublished trials and abstracts because our methodology required data available in full publications only. Meta-analysis based on individual data is considered by some authors as a gold standard (Stewart and Parmar, 1993). Systematic reviews of the literature should not be confused with meta-analyses of individual patient data. The first approach is based on fully published studies and provides an exhaustive and critical analysis on the topic with an adequate methodology based on the criteria of Mulrow (1987). The second approach is, in fact, a new study taking into account all performed studies on the topic, published or not, requiring individual data update by the investigators and is much more time-consuming. Nevertheless, as shown by our meta-analysis on the role of prophylactic cerebral irradiation in small-cell lung cancer (Meert *et al*, 2001), based on published data, we obtained the same results as in the meta-analysis based on individual data (Auperin *et al*, 1999).

Variations in survival results among the studies could be explained by the heterogeneity in methodologies used to stain and count microvessels in the tumours in addition to variation in patients population. The estimated vascularity in tissues sections can be significantly affected by variations in the applied methodology including pre-treatment and antibody use. The vessels in tumour samples can be identified by some different endothelial cell-specific antibodies: most often recognising factor VIII, CD31 or CD34 related antigen. Factor VIII (Von Willebrand's factor) was one of the first marker used for staining microvessels but it may be imprecise to quantify microvasculature for various reasons. Firstly, factor VIII is not expressed in all endothelial cells. The microvessels endothelial cells are less rich in Weibel-Palade bodies, which store factor VIII, than the endothelial cells of macrovessels (and the endothelial cells of neocapillaries may be activated by cytokines releasing their factor VIII stores). Secondly, factor VIII is also present in lymphatic endothelium and in platelets leading to a cross-reactivity with megakaryocytes, platelets and lymphatic endothelial cells. CD34, a heavily glycosylated transmembrane protein, is expressed on immature human haematopoietic precursor cells and is progressively lost during maturation. It is also present in the luminal endothelial membrane. CD34 is more sensitive and specific than factor VIII for staining endothelial cells induced by tumour neovascularisation (Tanigawa *et al*, 1996) but could also stain some lymphatic vessels. Only specific antibodies (i.e. LYVE 1, VEGF-C) can be used to detect lymphatics and not blood vessels. Anti-CD34 antibody seems to be more reliable in terms of specificity and reproducibility than monoclonal antibodies generated against other endothelial cell antigens (Tanigawa *et al*, 1996). In invasive breast cancer, CD34 has been shown to yield higher microvessel values than CD31 or factor VIII (Martin *et al*, 1997) and does not stain any tumour or inflammatory cells as CD31 or factor VIII. CD31 is a transmembrane glycoprotein highly expressed in mature and immature endothelium and its localisation at the endothelial cell junctions suggests an important role in transendothelial migration. CD31 is expressed during myelomonocytic cellular differentiation and consequently may cross-react with plasma cells, platelets, neutrophils, peripheral T cells and mantle zone B cells; endothelial staining can be easily differentiated on the basis of the morphological differences. JC70 antibody stains also CD31 positive lymphocytes that could be a prognostically important inflammatory component in lung cancer (Giatromanolaki *et al*, 1997). For some authors, CD31 seems to be the most sensitive marker for the endothelial cells and consistently stains more vessels than did factor VIII (Horak *et al*, 1992). An international consensus on the methodology and criteria of evaluation of microvessel density proposed that anti-CD31 monoclonal antibody should be the standard for microvessel assessment (Vermeulen *et al*, 1996) as it is superior on paraffin sections. But as CD34 has been shown to yield higher microvessel values than CD31 or factor VIII in breast cancer (Martin *et al*, 1997), it might be useful to combine CD34 and CD31 antibodies. In lung cancer, Offersen *et al* (2001) compared the staining with these three antibodies. He found that CD34 showed the best labelling of the endothelial cells and no background staining (data not shown). Yano *et al* (2000) found that correlation between factor VIII and CD34 staining for MVC was not strong and that staining for CD 34 significantly correlated with survival in adenocarcinoma but staining for factor VIII did not. Duarte *et al* (1998) reported that CD 31 did not predict survival in stage I NSCLC and did not correlate strongly with factor VIII which is correlated with lung cancer death. Giatromanolaki *et al* (1997) concluded that CD31 is sensitive for highlighting small, immature microvessels and is better correlated with nodal involvement and overall survival than factor VIII. Unfortunately, data were not sufficient to compare the three antibodies by a meta-analysis methodology.

Contradictory results in the literature may also be explained by variations in vascularity between areas in different sections from the same block or among blocks taken from the same tumour (de Jong *et al*, 1995) and by the methods used to measure vascularity (Pazouki *et al*, 1997). In large tumours, it could be necessary to examine multiple blocks in order to determine the overall vascularity of the tumour. Identifying the area of maximal microvessel density seems to be an important step in the counting method (Vermeulen *et al*, 1997) as tumour dissemination is more likely to occur at sites of high microvessel density. In lung cancer, the border between malignant and benign tissues is often blurred by atelectases, fibrosis and inflammatory cells, making the problem more difficult. The difficulty in recognising the vascular ‘hotspots’ may account for studies that failed to find an association between MVC and poor patients survival.

The technique used to count the microvessels is also different among the articles. Most of the studies used a technique similar to that proposed by Weidner *et al* (1991). The areas of highest neovascularization (‘hotspots’) is found by scanning the tumour sections at low power (40× and 100×) and then individual microvessels are counted on a 200× and 400× field. Each count is expressed as the highest number of microvessels identified within any 200× or 400× field. This technique is slow and laborious. A eye piece graticule (as a 25-point Chalkey graticule) has also been applied for vascular scoring in patients with NSCLC (Giatromanolaki *et al*, 1996a). In breast cancer, Fox *et al* (1995) showed that Chalkey counting is a rapid and objective method of quantifying tumour angiogenesis and gives independent prognostic information. A proposition of consensus identified the Chalkey method as slightly more objective (Vermeulen *et al*, 1996). We did not perform aggregation of the results in term of microvessel counting technique because the techniques were too heterogeneous.

Moreover, there is no standardised cut-off used for stratifying patients into high and low vascular groups. Some authors used the MVC median or the MVC mean and others the ‘best cut-off’, which is often arbitrary defined or chosen using multiple tests with a corresponding increase in the probability of founding a false positive results. The selection of the median value of the expression levels is a standard approach to analyse new prognostic factors, even if it may lead to some loss of information.

Assessment of tumour vascularity by immunohistochemistry on paraffin-embedded tissues can be easily performed in laboratory but standardisation of angiogenesis quantification is necessary in order to better define its prognostic value (Vermeulen *et al*, 1996) and to facilitate a routine use.

In conclusion, a high MVC, reflecting tumour neoangiogenesis, is a poor survival prognostic factor for NSCLC surgically treated patients. These results were based on an aggregation of data obtained by univariate survival analysis in retrospective trials. In order to become an useful prognostic factor, a standardisation of angiogenesis quantification is necessary and the present results need to be confirmed by an adequately designed prospective study with an appropriate multivariate analysis taking into account the classical well defined prognostic factors for lung cancer.
